# Physical Activity Levels in Kazakhstan: A Cross-Sectional Nationwide Study on Demographic, Socioeconomic, and Regional Factors

**DOI:** 10.3390/medicina61111913

**Published:** 2025-10-25

**Authors:** Anel Ibrayeva, Marat Shoranov, Talgat Muminov, Yerlan Ismoldayev, Shynar Tanabayeva, Ildar Fakhradiyev

**Affiliations:** 1 Department of Medicine, S.D. Asfendiyarov Kazakh National Medical University, Almaty 050000, Kazakhstan; 2 College of Medicine, Korea University, Seoul 02841, Republic of Korea

**Keywords:** physical activity, Kazakhstan, sedentary behavior, regional disparities, nationwide survey, WHO STEPS survey

## Abstract

*Background and Objectives*: Physical inactivity contributes to high rates of cardiovascular disease, diabetes, and obesity in Kazakhstan, yet national data remain scarce. The primary objective of this study was to assess the level of physical activity among the adult population of Kazakhstan. The secondary objectives were to identify demographic, socioeconomic, and regional factors associated with physical activity, to determine risk groups, and to develop recommendations aimed at increasing physical activity and improving public health. *Materials and Methods*: We conducted a cross-sectional nationwide survey (December 2021–May 2022) including 6720 adults aged 18–69 years from all 17 regions. Data were collected using the WHO STEPS questionnaire and the Global Physical Activity Questionnaire. *Results*: Overall, 19.7% of adults did not meet WHO recommendations. Average weekly activity was 7108 min, mainly from transport (42.1%) and work (28.9%). Men and young adults were more likely to be sufficiently active. Lower levels were observed among women, individuals over 35, married respondents, civil servants, students, and the unemployed. Significant regional and ethnic disparities were identified. *Conclusions*: One in five adults in Kazakhstan has insufficient physical activity. Targeted, multisectoral interventions are needed, with special focus on women, older adults, and urban populations.

## 1. Introduction

Physical activity is an integral part of a healthy lifestyle and plays a key role in the prevention of chronic diseases [1]. Physical inactivity is a significant risk factor for major non-communicable diseases [2,3,4], including ischemic heart disease [5], type 2 diabetes [6], chronic kidney disease [7], breast cancer [8], and colorectal cancer [9]. According to the 2019 Global Burden of Disease analysis, physical inactivity accounted for 7.8% of all deaths and 7.3% of disability-adjusted life years (DALYs) worldwide [10]. Globally, about 23% of adults do not meet recommended activity levels and, despite the WHO target of a 15% reduction by 2030 [11], current trends suggest this goal is unlikely to be achieved [12,13].

In Kazakhstan, the 2022 WHO Physical Activity Profile reports that 26% of men and 29% of women do not meet recommendations [14]; disparities observed already among children and adolescents by sex, family socioeconomic status, and place of residence indicate that inequalities in physical activity emerge early and likely persist into adulthood [15].

Insufficient activity leads to meaningful losses in life expectancy (the projected gain if inactivity were eliminated is ≈0.79 years for Kazakhstan) [16] and to economic costs: globally ~INT USD 520 billion across 2020–2030 [17], and in Kazakhstan about 0.34% of all health expenditures in 2013 [10]. Given the high prevalence of cardiovascular disease, diabetes, and obesity, increasing physical activity remains a key lever to reduce the burden on the health system and improve population quality of life [18,19].

Despite the obvious importance of this issue, studies on physical activity levels among the population of Kazakhstan are almost nonexistent. One exception is a study conducted in the Aral Sea region, but due to the small number of participants and the specific ecological and social conditions, it does not reflect the overall situation in the country [20].

In addition, pronounced regional differences in socioeconomic development, availability of sports infrastructure, and lifestyle warrant a more detailed examination of the factors shaping physical activity levels. The paucity of nationally representative data across demographic groups hinders the design of effective public health strategies for noncommunicable disease prevention in Kazakhstan. To address these gaps, this study provides the first nationally representative estimates of adult physical activity and sedentary behavior in Kazakhstan using standardized WHO STEPS/GPAQ-2 methods. We estimate national and region-specific prevalence; disaggregate activity by domains (work, transport, leisure); and quantify sociodemographic correlates with multivariable models to identify priority groups. These contributions provide a policy-relevant evidence base to support progress toward the WHO 2030 physical activity target in Kazakhstan.

The primary objective of this study was to assess the level of physical activity among the adult population of Kazakhstan. The secondary objectives were to identify demographic, socioeconomic, and regional factors associated with physical activity; to determine risk groups; and to develop recommendations aimed at increasing physical activity and improving public health.

## 2. Materials and Methods

### 2.1. Study Design

Kazakhstan is a Central Asian country with a population of approximately 20 million, and is administratively divided into 14 regions, three major cities of national significance (Astana, Almaty, and Shymkent), and 177 districts. Despite a low overall population density of six people per square kilometer, the majority of the population resides in urban areas.

This cross-sectional study, conducted between 1 December 2021, and 30 May 2022, included 6720 adults (50.1% men and 49.9% women) from all 14 regions and the three major cities.

Reporting follows the Strengthening the Reporting of Observational Studies in Epidemiology (STROBE) guideline for cross-sectional studies [21], and a completed STROBE checklist with page/line references is provided in the Supplementary Materials (Table S1).

### 2.2. Sampling Design

**Figure 1:**
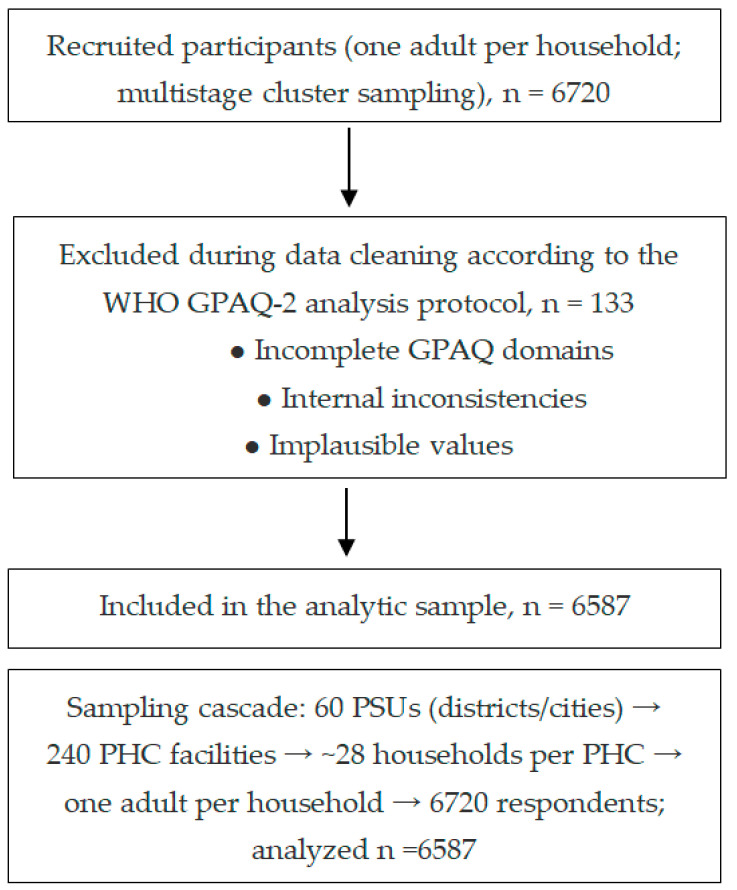
STROBE-compliant study flow diagram. PSU = primary sampling unit; PHC = primary healthcare facility.

The selection was based on data from the Bureau of National Statistics. The study targeted adults aged 18 to 69 years and employed a multistage cluster sampling method. Participants were categorized into five age groups: 18–24, 25–34, 35–44, 45–54, and 55–69 years. The sample size was determined using the WHO STEPS sample size calculator, assuming a hypothesized prevalence of risk factors at 50%, a standard error of 5%, a design effect coefficient of 1.5, and a projected response rate of 70%. The calculated minimum sample size was 6585, but 6720 participants were recruited to ensure adequate representation (Figure 1).

To select participants, the sampling process involved three stages. First, primary sampling units (PSUs), comprising districts and cities, were selected proportionally from all economic regions of Kazakhstan, resulting in 60 selected PSUs out of 266 possible administrative units. Second, within each selected PSU, 4 primary healthcare (PHC) facilities were randomly chosen from a national registry, resulting in a total of 240 selected PHC facilities. The selection process was based on the probability proportional to the population served. Finally, the tertiary sampling units, households and respondents, were chosen. The number of required households per PHC facility was calculated as approximately 28, leading to a total of 6720 respondents. A list of households served by the selected PHC facilities was obtained, and random selection was conducted using the Kish methodology to ensure fair representation by gender and age.

Of 6720 respondents recruited, 6587 remained for analysis after data cleaning in accordance with the WHO GPAQ-2 analysis protocol; records with incomplete GPAQ domains, internal inconsistencies, or implausible values were excluded.

### 2.3. Data Collection

Prior to data collection, research teams received training on interview techniques and physical and biochemical measurements. Additionally, they completed good clinical practice (GCP) training and obtained certification, ensuring adherence to ethical and methodological standards in research. Data were collected through face-to-face interviews, followed by physical and biochemical measurements conducted on the same day.

### 2.4. Data Variables

The study followed the standardized WHO STEPwise approach [22]. In the first stage, data on sociodemographic characteristics, including age, sex, ethnicity, place of residence, education level, marital status, and occupation, were collected, along with information on behavioral risk factors such as smoking and physical activity. The second and third stages included physical measurements and blood sample collection.

Body mass index (BMI) was calculated and categorized into underweight (<18.5 kg/m^2^), normal weight (18.5–24.9 kg/m^2^), overweight (25–29.9 kg/m^2^), and obesity (≥30 kg/m^2^).

### 2.5. Physical Activity Measurement

Physical activity was assessed using the Global Physical Activity Questionnaire (GPAQ, version 2.0) [23], which covers three domains: work, transport, and leisure. We defined meeting the WHO recommendations according to the 2020 Guidelines and operationalized MVPA as MET-minutes/week derived from GPAQ domains. Sedentary time was treated as an independent exposure and measured with the GPAQ item “How much time do you usually spend sitting or reclining on a typical day?”, reported in minutes/day and—for interpretation—categorized using established cut-offs. For work and leisure, participants reported vigorous and moderate activities; for transport, only moderate-intensity activity (walking or cycling) was captured. For each affirmative domain, participants provided the number of days per week and the average duration per day.

Physical activity data were processed according to the WHO GPAQ Analysis Guide [23,24]. Domain-specific MET-minutes/week were computed as days × minutes × MET value (8 METs for vigorous and 4 METs for moderate activity, including walking), and total physical activity was the sum across domains. Following common practice in GPAQ analyses, we defined activity levels using MET-minute thresholds only: high (≥3000 MET-minutes/week from any combination of walking, moderate, or vigorous activity, or ≥1500 MET-minutes/week from vigorous activity alone), moderate (600–2999 MET-minutes/week), and low (<600 MET-minutes/week).

### 2.6. Bias

Potential sources of bias included self-reported physical activity and sedentary time (recall and social desirability bias), selection related to recruitment through primary healthcare facilities, and non-response. We minimized bias through a multistage probability sampling design covering all regions with urban–rural strata; standardized WHO STEPS and GPAQ-2 instruments; centralized interviewer training and GCP certification; and pre-specified data-cleaning rules according to the WHO GPAQ Analysis Guide (exclusion of incomplete domains, internal inconsistencies, and implausible values). Physical and biochemical measurements were taken using calibrated equipment following STEPS protocols. Analyses used a complete-case approach without imputation; the proportion of missing covariate data did not exceed 1–2%. Residual bias is acknowledged in the Strengths and Limitations section.

### 2.7. Statistical Analysis

Data were initially entered into Microsoft Excel and subsequently analyzed using SPSS software (version 24.0, IBM Corp., Armonk, NY, USA). The Kolmogorov–Smirnov test was applied to assess data distribution. Continuous variables were compared using the Student’s *t*-test, while categorical variables were analyzed using Pearson’s chi-square (χ^2^) test. Proportions were reported with 95% confidence intervals (CIs) to ensure accurate interpretation of population level estimates. Analyses used a complete-case approach; no imputation was performed.

Both unadjusted (crude) and adjusted binary logistic regression models were used to examine associations between physical activity levels (dependent variable) and sociodemographic and behavioral factors (independent variables). The adjusted model included all independent variables as covariates to control for potential confounding. Results were presented as odds ratios (ORs) with 95% CIs. Associations were considered statistically significant if the CI did not include 1.0 and *p*-values were less than 0.05. To illustrate regional variations in physical activity across Kazakhstan, a visual map was created using Datawrapper (https://www.datawrapper.de/).

## 3. Results

### 3.1. Structure of Physical Activity

The average total duration of physical activity was 1361.9 min per week or approximately 194.6 min per day (Table 1). Transport accounted for the largest share of total daily activity (50.1%), followed by work (32.2%), while leisure contributed the smallest share (16.9%). Among men, the main source was work (121.8 ± 204.1 min/day), whereas among women it was transport (67.2 ± 92.6 min/day). Leisure-time activity was minimal in both sexes, particularly among women (17.9 ± 49.3 min/day). At the national level, 56.3% reported no work activity, 24.5% no transport activity, 58.1% no leisure-time activity, and 64.2% no vigorous activity (Table 2); sex differences were significant across domains (χ^2^, *p* < 0.001).

### 3.2. Characteristics of Respondents with a Sedentary Behavior

**Figure 2:**
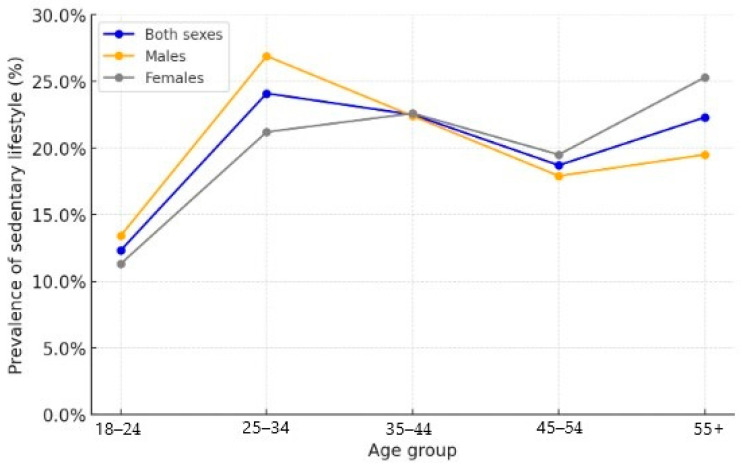
Prevalence of sedentary lifestyle by age and sex.

The highest proportion of people with low physical activity were in the 25–34 age group (24.10%, 95% CI: 22.70–25.60), and the lowest—among young people aged 18–24 (12.30%, 95% CI: 11.30–13.50). In the younger age groups, men showed a higher level of sedentary behavior compared to women, whereas among individuals aged 55 and older, this indicator was higher among women (25.30%, 95% CI: 23.30–27.50) than men (19.50%, 95% CI: 17.70–21.40) (Table 3, Figure 2).

### 3.3. Gender Differences in Physical Activity

Men demonstrated higher physical activity levels than women: 56.9% (95% CI: 55.2–58.6) vs. 47.2% (45.5–49.0) classified as high, whereas low activity was more common among women (21.6%, 20.2–23.0) than men (17.8%, 16.5–19.1); the proportion at moderate level was also higher in women (31.1%, 29.6–32.7) than men (25.3%, 23.9–26.8) (Table 4).

### 3.4. Age-Specific Characteristics of Physical Activity

The highest level of physical activity was observed in the 25–34 (57.40%, 95% CI: 55.00–59.90) and 18–24 (55.50%, 95% CI: 52.20–58.70) age groups. Starting from the age of 35, physical activity declined, reaching minimum values among individuals over 55 years of age.

### 3.5. Socioeconomic Factors

Participants with secondary education had the highest share classified as high physical activity (54.5%, 95% CI: 52.5–56.6), followed by those with higher education (51.0%, 49.5–52.5), while the lowest was among participants with primary education (45.9%, 35.1–56.8). Married participants more frequently showed a low level of activity (21.90%, 95% CI: 20.70–23.20), whereas single individuals more often displayed a high level (57.30%, 95% CI: 54.80–59.80). Among professions, private sector employees were the most active (55.00%, 95% CI: 53.20–56.70), followed by civil servants (51.00%, 95% CI: 48.60–53.40), and the unemployed (44.70%, 95% CI: 39.90–49.50) were the least active.

### 3.6. Ethnic and Regional Differences

**Figure 3:**
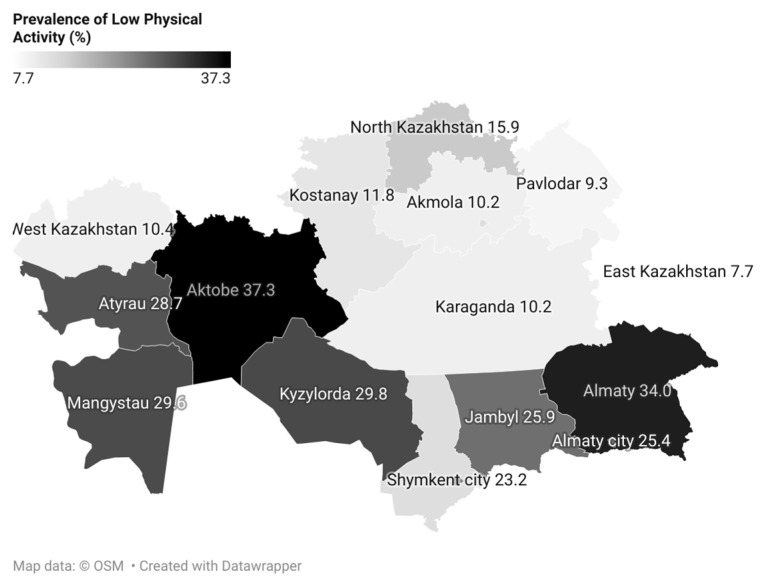
Regional differences in the prevalence of low physical activity in Kazakhstan.

Among ethnic groups, the share classified as low physical activity was higher among Kazakhs (22.3%, 95% CI: 21.1–23.6), whereas Russians had the highest share classified as high (58.9%, 56.5–61.4). Regionally, the highest proportions with high physical activity were observed in the East Kazakhstan region (67.6%, 63.2–71.9), Akmola region (65.0%, 59.9–70.1), Astana city (63.5%, 59.1–68.0), the Kostanay region (63.1%, 57.9–68.3), and Karaganda region (62.8%, 58.3–67.3). Figure 3 maps the prevalence of low physical activity across regions; the highest levels of low activity were observed in the Aktobe region (37.3%, 95% CI: 32.1–42.5), Almaty region (34.0%, 30.0–37.9), and Kyzylorda region (29.8%, 24.9–34.8).

### 3.7. Relationship of Physical Activity with BMI and Harmful Habits

A high level of physical activity was more often observed among participants with normal body weight (54.50%, 95% CI: 52.60–56.50), while among overweight individuals, a low activity level was more frequent (20.50%, 95% CI: 19.30–21.80). Among individuals classed as underweight, a moderate level of physical activity was more common (37.90%, 95% CI: 31.30–44.40).

Low physical activity was more often observed among non-smokers (21.1%, 95% CI: 20.0–22.2) compared to smokers (17.8%, 95% CI: 15.8–20.0); the differences were statistically significant (*p* = 0.001). In the smoker group, men predominated (79.4%), whereas in the non-smoker group, women were more prevalent (56.0%). The highest proportion of smokers with high physical activity was found in the 25–34 age group (31.0%) and 35–44 age group (27.6%). Among non-smokers, a high level of physical activity was also more common in the 25–34 age group (25.3%). At the same time, among non-smokers, the proportion of individuals with low physical activity increased with age, especially among respondents older than 45 years.

### 3.8. Factors Associated with Meeting WHO Recommendations

**Figure 4:**
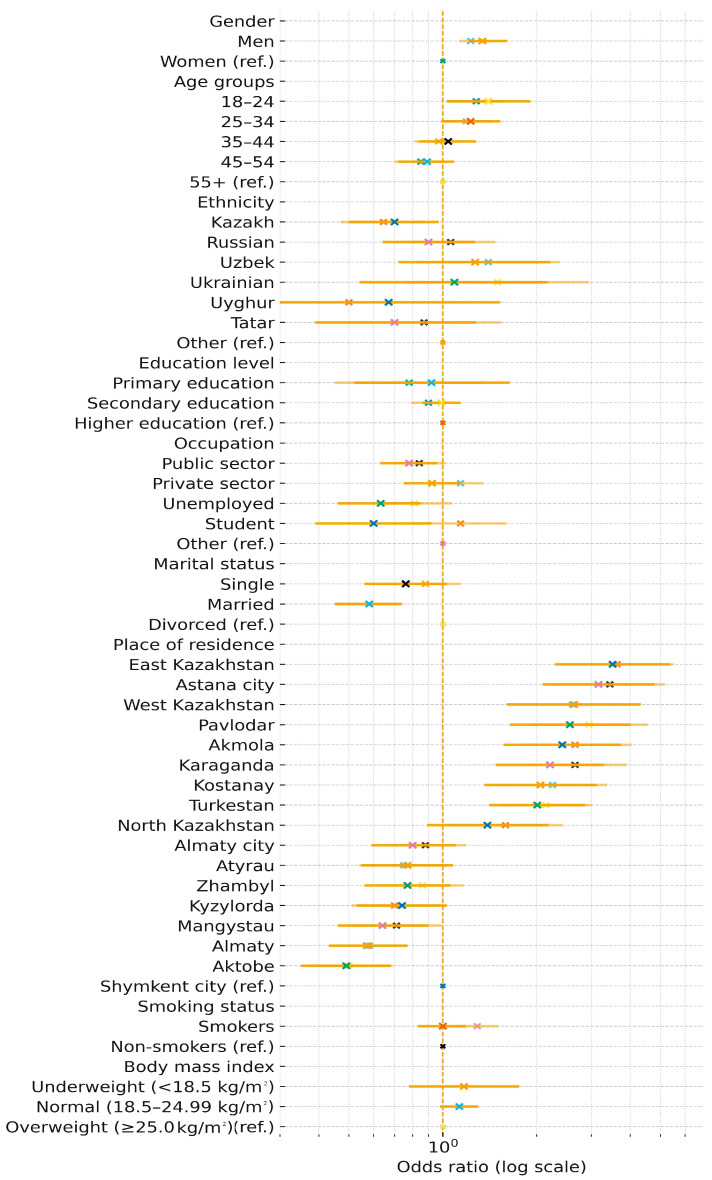
Determinants of meeting WHO physical activity recommendations (adjusted odds ratios).

According to the adjusted logistic regression, men were 34% more likely to meet WHO recommendations than women (AOR = 1.34; 95% CI: 1.21–1.61; *p* < 0.001) (Table 5, Figure 4). The highest odds were observed among adults aged 18–24 versus those 55+ (AOR = 1.40; 95% CI: 1.03–1.91; *p* = 0.032). Kazakh ethnicity was associated with lower odds (AOR = 0.70; 95% CI: 0.50–0.97; *p* = 0.035). Married respondents had reduced odds relative to divorced (AOR = 0.58; 95% CI: 0.45–0.74; *p* < 0.001).

The highest level of physical activity was recorded among residents of the East Kazakhstan region (OR = 3.51; 95% CI: 2.30–5.36) and Astana (OR = 3.17; 95% CI: 2.09–4.80), where the likelihood of meeting WHO criteria was 3–3.5 times higher than in Shymkent. Meanwhile, the lowest level of physical activity was observed in Almaty (OR = 0.58; 95% CI: 0.43–0.77), the Aktobe region (OR = 0.49; 95% CI: 0.36–0.69), and the Almaty region (OR = 0.81; 95% CI: 0.59–1.10), where the OR values indicated a high prevalence of sedentary behavior.

## 4. Discussion

In our study, the level of physical inactivity among the adult population of Kazakhstan was 19.7%, which is higher than in Kyrgyzstan (11.4%) and Uzbekistan (16.4%), but lower than in Tajikistan (28.3%), according to Whiting et al. [25]. Thus, Kazakhstan occupies an intermediate position among the Central Asian countries, which may reflect differences in the degree of urbanization, employment patterns, and the accessibility of infrastructure for physical activity.

Our results show clear gender differences in both the level and domains of physical activity. Consistent with prior work [19,26], men are more active overall and accrue a larger share of activity through work, likely reflecting more physically demanding jobs and persistent gender roles. Women accumulate more transport-related activity, in line with evidence that they more often walk or cycle [27]; in Kazakhstan this mirrors mobility patterns—men rely more on private cars, whereas women more often use public transport and walking.

Leisure-time physical activity was the least common domain of activity for both sexes, but men spent significantly more time on active leisure than women. These differences may be due to social and cultural factors, as traditional family stereotypes in Kazakhstan often discourage women from engaging in sports or active recreation, leading them to spend more of their free time on household and family responsibilities. This pattern of lower female participation in leisure exercise is consistent with findings from other studies [28,29]. Notably, leisure-time physical activity has a more pronounced positive effect on health compared to occupational physical activity. According to Quinn et al. [30], recreational exercise contributes to improved cardiovascular health, reduced stress levels, and better quality of life, whereas excessive physical activity related to work can negatively impact health due to high physical strain. Therefore, the gender gap in active leisure highlights the need to develop programs that promote leisure-time exercise—especially among women—through community sports, fitness clubs, and other initiatives that encourage active recreation in their free time.

Age was another important factor associated with physical activity levels. The highest proportion of individuals not meeting the WHO recommendations was observed in the 35–44 age group, likely due to peak professional workloads and family responsibilities in mid-life. This is consistent with Panter et al. [31], who noted that many middle-aged adults face time limitations that reduce their physical activity. In contrast, younger adults (18–24 years) had the greatest likelihood of meeting the WHO recommendations, which can be explained by a relative abundance of free time and greater involvement in sports and active recreation. This finding aligns with the observations of Guthold et al. [19], who reported high activity levels among youth, particularly in countries where educational institutions provide access to sports infrastructure.

Certain occupational and social factors were also linked to lower physical activity. Civil servants, the unemployed, and students were less likely to meet the WHO criteria, which may be due to the sedentary nature of office work (for many civil servants), financial or access constraints limiting sports participation for the unemployed, and heavy academic workloads leaving students with little leisure time for exercise. These observations are in line with international studies on how sedentary jobs and low awareness of physical activity benefits contribute to inactivity [32,33]. Similarly, married respondents were less likely to meet the WHO recommendations, possibly because of increased family responsibilities and a reallocation of time—with childcare and household duties taking precedence over personal exercise.

There were notable regional and ethnic disparities in physical activity levels, which appear to be interrelated. Ethnic Kazakhs exhibited lower overall activity levels, which may be linked to their high concentration in the urbanized southern regions of the country. Indeed, some of the lowest physical activity indicators were recorded in major southern areas such as Almaty city and the Almaty region. These regions are characterized by dense populations, heavy traffic, and greater urbanization, all of which can limit opportunities for an active lifestyle. For example, Almaty—Kazakhstan’s largest metropolis—experiences severe traffic congestion (approximately one million vehicles on the roads daily, including about 400,000 commuters from the surrounding region) that results in long commuting times and leaves residents with less time for exercise. Additionally, the hot summer climate in the south may reduce motivation for outdoor physical activity, further predisposing the population to a sedentary behavior. In contrast, areas with traditionally higher Russian populations, such as the northern and eastern oblasts (e.g., Karaganda, East Kazakhstan), demonstrated a higher level of physical activity. These regions have lower population density and less traffic congestion, which likely increases the share of active modes of transportation like walking and cycling.

In our study, a low level of physical activity was more common among non-smokers than smokers. This counterintuitive finding is likely explained by demographic differences between the groups: the smoker group contained predominantly men and working-age adults, who tend to have higher activity levels, whereas the non-smoker group included more women and older individuals, who generally were less active. This example highlights the need to consider underlying demographic factors when analyzing the relationship between lifestyle behaviors (like smoking) and physical activity.

Body weight showed only a weak association with meeting physical activity recommendations. Although individuals who achieved the WHO-recommended activity levels included a slightly higher proportion of those with normal BMI (and fewer who were overweight) compared to inactive individuals, these differences did not reach statistical significance. This confirms that physical activity is only one of the factors influencing body weight and that a comprehensive approach—including proper diet and attention to individual physiological characteristics—is required for effective weight management [34].

Older adults emerged as a group requiring special attention. There is a need for programs targeting the elderly that include group exercises, guided wellness walks, senior gymnastics, and dancing—activities that help maintain health and also provide social engagement. It is especially important to reduce sedentary time among older women, as this subgroup showed the highest level of physical inactivity in our study: among adults aged 55 and above, 25.3% of women were insufficiently active, compared to 19.5% of men. This finding underscores the need for targeted interventions that take into account both age and gender differences [35,36]. In Kazakhstan, “Active Longevity” centers have already been established to promote physical activity and social involvement among the elderly population [37]. However, to reach a larger number of seniors—especially in areas with limited infrastructure—further expansion of such programs is needed.

### Perspectives for Clinical Practice in a Public Health Context

Overall, our findings underscore the need for targeted strategies to raise physical activity across population subgroups and for coordinated clinical and public health action. At the population level, priorities include reducing sedentary behavior by introducing active workplace practices (short exercise breaks, ergonomic workstations), regular movement breaks, and education campaigns; developing convenient and safe infrastructure for active travel (well-maintained sidewalks, pedestrian zones, bike lanes, and end-of-trip facilities); and community initiatives that stimulate leisure-time activity with targeted engagement of women and older adults. In parallel, primary care should normalize routine screening and brief, goal-oriented counseling on physical activity with documentation in the electronic health record, and use standardized referral pathways to community programs and “safe” activity modules for patients with chronic conditions; clinical networks and low-burden digital supports (SMS/app prompts, wearables where feasible) can help sustain adherence and monitor equity of reach across sex, age, region, and occupation [38,39,40].

These directions are operationalized in the recent international agenda (2022–2024): the MOVING framework integrates six action areas—school- and community-based opportunities, workplace initiatives, supportive physical environments, active-transport infrastructure, public communication strategies, and integration of physical-activity assessment and counseling into healthcare—providing a cross-sector implementation roadmap [41]. Within healthcare systems, the “It’s Time to Move” initiative emphasizes standardized metrics and documentation of physical activity, light-touch digital solutions, and public–private partnerships to accelerate scale-up [42]. Post-COVID evidence shows a sustained shift toward home- and community-based, online, and app-supported formats [43]; when access and digital literacy barriers are addressed, these models extend reach to women and older adults—groups with lower leisure-time activity in our data [44]. Our patterns also confirm that activity levels are shaped not only by individual choice but by structural factors (work organization, transport systems, the built environment, climate); accordingly, policy should pair population-level interventions with targeted support for women, older adults, mid-life workers, and residents of low-performing regions. In practice, sustaining higher activity levels is constrained by limited financial/infrastructural resources, competing family and work responsibilities, and cultural norms [45,46]—especially for women and older adults—which should be explicitly addressed in implementation planning and scale-up.

## 5. Conclusions

This nationally representative study provides the first comprehensive picture of adult physical activity in Kazakhstan and shows that nearly one in five adults does not meet WHO recommendations. Marked disparities by sex, age, occupation, marital status, and region persist, with particularly low leisure-time activity among women and high sedentary time in older adults. These patterns indicate that activity is shaped not only by individual choice but also by structural conditions in work organization, transport systems, and the built environment.

In practical terms, the results support a shift from individual messaging to a coordinated clinical–public health response. In primary care, routine screening and brief, goal-oriented counseling should be normalized, documented in the electronic health record, and linked to standardized referral pathways to community programs and safe activity modules for patients with chronic conditions; clinical networks and light-touch digital tools should be used to sustain adherence and monitor equity. From a public health perspective, priorities include workplace programs for sedentary workers (especially in the public sector), expansion of protected cycling and pedestrian infrastructure with end-of-trip facilities, and targeted, affordable leisure-time offers for women and older adults, with regional tailoring for southern metropolitan areas. Methodologically, future waves should add objective measures in subsamples, strengthen harmonized reporting, and evaluate scalable interventions using pragmatic or quasi-experimental designs with equity and cost-effectiveness assessments to inform implementation at scale.

These recommendations align with national priorities in Healthy Nation 2021–2025, the national digital health agenda (electronic health record prompts/templates and standardized referral pathways), and urban mobility programs (protected cycling and pedestrian infrastructure with end-of-trip facilities). Embedding routine physical activity screening and brief counseling into primary-care clinical standards, adopting workplace physical activity standards for civil service organizations, and co-financing active-mobility infrastructure with local authorities provide near-term policy levers for equitable implementation and scale-up.

## 6. Strengths and Limitations

This study has some strengths and limitations. A nationally representative multistage, stratified cluster sampling approach was used, ensuring robust representativeness across all 17 regions of Kazakhstan, including both urban and rural populations. The large sample size (*n* = 6720) included diverse sociodemographic groups, enhancing the statistical power and generalizability of the findings. The use of the standardized WHO STEPwise and GPAQ-2 methodologies allows comparability with international studies and ensures high methodological quality. The cross-sectional design limits the ability to establish causal relationships, meaning all observed associations should be interpreted as correlational rather than causal. Physical activity was self-reported using the GPAQ questionnaire, which may introduce recall bias or social desirability bias. Objective tools such as accelerometers were not used due to the large sample size and logistical constraints. Recruitment via primary healthcare facilities may have introduced selection bias—PHC attendees can differ from non-attenders in health status and lifestyle—so generalizability, particularly to hard-to-reach rural or otherwise marginalized groups, may be limited despite the stratified design.

## Figures and Tables

**Table 1:** Time-based and compositional measures of physical activity (mean ± SD).

Measure	Men (*n* = 3350)	Women (*n* = 3237)	Both Sexes (*n* = 6587)	*p*-Value
Total physical activity (min)				
Total per week (min/week)	1513.7 ± 1831.9	1205.2 ± 1609.8	1361.9 ± 1732.9	0.0001
Total per day (min/day)	216.2 ± 261.7	172.2 ± 229.9	194.6 ± 247.6	0.0001
Daily minutes by domain (min/day)				
Work	121.8 ± 204.1	86.9 ± 174.3	104.7 ± 190.8	0.0001
Transport	64.6 ± 92.2	67.2 ± 92.6	65.9 ± 92.4	0.0001
Leisure	29.8 ± 60.1	17.9 ± 49.3	23.9 ± 55.4	0.0001
Composition of total daily activity (% of total)				
Work	36.1 ± 37.9	28.1 ± 35.9	32.2 ± 37.1	0.0001
Transport	43.4 ± 36.6	58.5 ± 37.8	50.1 ± 37.9	0.0001
Leisure	20.4 ± 28.6	13.4 ± 24.3	16.9 ± 26.8	0.0001
Sedentary time (min/day)				
Mean sedentary	288.4 ± 170.7	282.2 ± 171.6	285.3 ± 171.2	0.321

SD: standard deviation.

**Table 2:** Prevalence-based measures of physical activity (%; 95% CI).

Measure	Men	Women	Both Sexes	χ^2^	*p*-Value
Work-related activity					
Did no work activity	51.7 (50.0–53.4)	61.0 (59.3–62.6)	56.3 (55.1–57.5)	58.3	0.0001
Did work activity	48.3 (46.6–50.0)	39.0 (37.4–40.7)	43.7 (42.5–44.9)	—	—
Transport-related activity					
Did no transport activity	27.7 (26.2–29.2)	21.1 (19.8–22.6)	24.5 (23.4–25.5)	38.5	0.0001
Did transport activity	72.3 (70.8–73.8)	78.9 (77.4–80.2)	75.5 (74.5–76.6)	—	—
Leisure-time activity					
Did no recreation activity	51.9 (50.2–53.6)	64.6 (62.9–66.2)	58.1 (56.9–59.3)	112.5	0.0001
Did recreation activity	48.1 (46.4–49.8)	35.4 (33.8–37.1)	41.9 (40.7–43.1)	—	—
Vigorous activity					
Did no vigorous activity	55.4 (53.7–57.1)	73.1 (71.5–74.6)	64.2 (63.1–65.4)	226.9	0.0001
Did vigorous activity	44.6 (42.9–46.3)	26.9 (25.4–28.5)	35.8 (34.6–36.9)	—	—

CI: confidence interval.

**Table 3:** Characteristics of respondents with a sedentary behavior, % (95% CI).

Age Categories	Both Sexes	Men	Women
18–24	12.3 (11.3–13.5)	13.4 (11.8–15.0)	11.3 (9.8–12.8)
25–34	24.1 (22.7–25.6)	26.9 (24.8–29.0)	21.2 (19.3–23.3)
35–44	22.5 (21.1–23.9)	22.4 (20.4–24.4)	22.6 (20.7–24.7)
45–54	18.7 (17.4–20.1)	17.9 (16.2–19.8)	19.5 (17.7–21.5)
55+	22.3 (21.0–23.8)	19.5 (17.7–21.4)	25.3 (23.3–27.5)

CI: confidence interval.

**Table 4:** Distribution of physical activity levels by sociodemographic characteristics, % (95% CI).

Variables	Low Physical Activity	Moderate Physical Activity	High Physical Activity	χ^2^	*p*-Value
Total	19.7 (18.7–20.7)	28.2 (27.1–29.3)	52.1 (50.9–53.4)	-	-
Gender					
Male	17.8 (16.5–19.1)	25.3 (23.9–26.8)	56.9 (55.2–58.6)	61.6	0.0001
Female	21.6 (20.2–23.0)	31.1 (29.6–32.7)	47.2 (45.5–49.0)		
Age groups					
18–24	16.5 (14.1–18.9)	28.0 (25.1–30.9)	55.5 (52.2–58.7)	42.9	0.0001
25–34	17.5 (15.6–19.4)	25.1 (23.0–27.3)	57.4 (55.0–59.9)		
35–44	20.7 (18.7–22.7)	29.8 (27.5–32.2)	49.5 (47.0–52.0)		
45–54	23.0 (20.7–25.3)	27.4 (25.0–29.9)	49.6 (46.8–52.3)		
55+	20.2 (18.0–22.3)	30.7 (28.3–33.1)	49.1 (46.4–51.8)		
Education					
Primary education	23.0 (13.5–32.4)	31.1 (20.3–41.9)	45.9 (35.1–56.8)	21.3	0.0001
Secondary education	20.7 (19.1–22.4)	24.7 (23.0–26.5)	54.5 (52.5–56.6)		
Higher education	19.1 (17.9–20.2)	30.0 (28.6–31.4)	51.0 (49.5–52.5)		
Marital status					
Single	15.6 (13.8–17.4)	27.1 (24.9–29.3)	57.3 (54.8–59.8)	50.3	0.0001
Married	21.9 (20.7–23.2)	28.2 (26.9–29.5)	49.9 (48.4–51.4)		
Divorced	14.0 (11.4–16.8)	30.6 (27.1–34.1)	55.3 (51.5–59.1)		
Occupation					
Public sector	22.6 (20.6–24.6)	26.4 (24.3–28.6)	51.0 (48.6–53.4)	39.2	0.0001
Private sector	17.8 (16.5–19.2)	27.2 (25.7–28.8)	55.0 (53.2–56.7)		
Unemployed	23.2 (19.1–27.3)	32.1 (27.8–36.7)	44.7 (39.9–49.5)		
Student	17.7 (13.5–22.2)	28.1 (22.9–33.3)	54.2 (48.3–60.1)		
Other	19.8 (17.6–22.2)	31.8 (29.1–34.5)	48.3 (45.5–51.2)		
Ethnicity					
Kazakh	22.3 (21.1–23.6)	28.5 (27.1–29.8)	49.2 (47.7–50.7)	80.1	0.0001
Russian	14.9 (13.1–16.7)	26.2 (24.0–28.4)	58.9 (56.5–61.4)		
Uzbek	11.7 (7.6–16.2)	37.6 (31.0–44.2)	50.8 (43.7–57.9)		
Ukrainian	11.0 (5.5–17.4)	28.4 (20.2–36.7)	60.6 (51.4–69.7)		
Uyghur	27.0 (13.5–40.5)	29.7 (16.2–45.9)	43.2 (27.0–59.5)		
Tatar	17.5 (10.5–24.6)	28.1 (20.2–36.8)	54.4 (45.6–63.2)		
Other ethnicity	15.7 (11.9–19.7)	27.6 (22.9–32.6)	56.7 (51.4–62.1)		
Region					
Astana city	8.1 (5.5–10.6)	28.4 (24.2–32.6)	63.5 (59.1–68.0)	543.8	0.0001
Almaty city	25.4 (21.7–29.0)	36.0 (32.0–40.0)	38.7 (34.6–42.7)		
Akmola	10.2 (6.9–13.4)	24.9 (20.2–29.5)	65.0 (59.9–70.1)		
Aktobe	37.3 (32.1–42.5)	26.1 (21.3–30.8)	36.7 (31.5–41.9)		
Almaty	34.0 (30.0–37.9)	33.6 (29.7–37.6)	32.4 (28.5–36.3)		
Atyrau	28.7 (23.8–33.6)	14.6 (10.8–18.5)	56.7 (51.3–62.1)		
West Kazakhstan	10.4 (6.4–14.4)	33.8 (27.6–40.0)	55.9 (49.3–62.4)		
Zhambyl	25.9 (21.8–30.0)	23.8 (19.8–27.8)	50.3 (45.7–55.0)		
Karaganda	10.2 (7.3–13.0)	27.1 (22.9–31.2)	62.8 (58.3–67.3)		
Kostanay	11.8 (8.3–15.3)	25.1 (20.4–29.7)	63.1 (57.9–68.3)		
Kyzylorda	29.8 (24.9–34.8)	23.7 (19.1–28.3)	46.5 (41.0–51.9)		
Mangystau	29.6 (24.7–34.5)	24.8 (20.1–29.4)	45.6 (40.3–51.0)		
Turkestan	12.3 (9.5–15.2)	33.1 (29.0–37.2)	54.6 (50.3–58.9)		
Pavlodar	9.3 (6.2–12.4)	31.0 (26.1–36.0)	59.7 (54.4–65.0)		
North Kazakhstan	15.9 (11.1–20.7)	27.3 (21.4–33.2)	56.8 (50.3–63.4)		
East Kazakhstan	7.7 (5.2–10.1)	24.8 (20.8–28.8)	67.6 (63.2–71.9)		
Shymkent city	23.2 (19.2–27.1)	32.3 (27.9–36.6)	44.5 (39.9–49.2)		
Smoking status					
Smokers	17.8 (15.8–20.0)	22.7 (20.4–25.0)	59.5 (56.8–62.2)	36.4	0.001
Non-smokers	21.1 (20.0–22.2)	28.7 (27.5–29.9)	50.2 (48.8–51.5)		
Body Mass Index					
Underweight (< 18.5 kg/m^2^)	17.2 (12.1–22.7)	37.9 (31.3–44.4)	44.9 (37.9–52.0)	18.0	0.001
Normal (18.5–24.9 kg/m^2^)	18.6 (17.1–20.1)	26.8 (25.1–28.5)	54.5 (52.6–56.5)		
Overweight (≥25.0 kg/m^2^)	20.5 (19.3–21.8)	28.6 (27.2–30.0)	50.9 (49.3–52.5)		

CI: confidence interval. Low: <600 MET-min/week; moderate: 600 to <3000 MET-min/week; high: ≥3000 MET-min/week.

**Table 5:** Logistic regression of factors associated with meeting WHO physical activity recommendations.

Variable	Unadjusted OR (95% CI)	*p*-Value	Adjusted OR (95% CI)	*p*-Value
Gender				
Men	1.23 (1.13–1.44)	0.0001	1.34 (1.21–1.61)	0.0001
Women	1 (Reference)	-	1 (Reference)	-
Age groups				
18–24	1.28 (1.03–1.60)	0.028	1.40 (1.03–1.91)	0.032
25–34	1.19 (0.99–1.44)	0.060	1.23 (0.99–1.53)	0.053
35–44	0.97 (0.81–1.17)	0.761	1.04 (0.84–1.28)	0.687
45–54	0.85 (0.70–1.02)	0.082	0.89 (0.72–1.09)	0.278
55+	1 (Reference)	-	1 (Reference)	-
Ethnicity				
Kazakh	0.645 (0.47–0.88)	0.006	0.70 (0.50–0.97)	0.035
Russian	1.06 (0.76–1.48)	0.711	0.90 (0.64–1.27)	0.567
Uzbek	1.40 (0.82–2.38)	0.207	1.27 (0.72–2.22)	0.398
Ukrainian	1.50 (0.76–2.94)	0.235	1.09 (0.54–2.17)	0.806
Uyghur	0.50 (0.22–1.10)	0.086	0.67 (0.29–1.53)	0.351
Tatar	0.87 (0.49–1.54)	0.642	0.70 (0.39–1.28)	0.256
Other	1 (Reference)	-	1 (Reference)	-
Education level				
Primary education	0.78 (0.45–1.36)	0.396	0.92 (0.52–1.64)	0.796
Secondary education	0.90 (0.79–1.02)	0.104	0.99 (0.86–1.14)	0.913
Higher education	1 (Reference)	-	1 (Reference)	-
Occupation				
Public sector	0.84 (0.70–1.02)	0.082	0.78 (0.63–0.96)	0.023
Private sector	1.14 (0.96–1.35)	0.129	0.923 (0.75–1.13)	0.444
Unemployed	0.81 (0.62–1.07)	0.148	0.632 (0.46–0.85)	0.003
Student	1.14 (0.82–1.60)	0.417	0.60 (0.39–0.92)	0.021
Other	1 (Reference)	-	1 (Reference)	-
Marital status				
Single	0.88 (0.68–1.14)	0.345	0.76 (0.56–1.03)	0.077
Married	0.58 (0.46–0.73)	0.0001	0.58 (0.45–0.74)	0.0001
Divorced	1 (Reference)	-	1 (Reference)	-
Place of residence				
Astana city	3.44 (2.29–5.17)	0.000	3.16 (2.09–4.79)	0.0001
Almaty city	0.88 (0.66–1.19)	0.427	0.80 (0.59–1.10)	0.180
Akmola	2.66 (1.75–4.04)	0.0001	2.42 (1.57–3.72)	0.0001
Aktobe	0.50 (0.37–0.69)	0.0001	0.49 (0.35–0.68)	0.0001
Almaty	0.58 (0.44–0.77)	0.0001	0.57 (0.43–0.77)	0.0001
Atyrau	0.75 (0.54–1.04)	0.086	0.77 (0.55–1.08)	0.135
West Kazakhstan	2.61 (1.60–4.24)	0.0001	2.64 (1.61–4.32)	0.0001
Zhambyl	0.86 (0.63–1.17)	0.357	0.77 (0.56–1.06)	0.109
Karaganda	2.66 (1.82–3.90)	0.0001	2.21 (1.48–3.29)	0.0001
Kostanay	2.25 (1.51–3.37)	0.0001	2.06 (1.36–3.12)	0.001
Kyzylorda	0.70 (0.51–0.98)	0.038	0.74 (0.53–1.03)	0.075
Mangystau	0.71 (0.51–0.99)	0.044	0.64 (0.46–0.90)	0.011
Turkestan	2.14 (1.52–3.02)	0.0001	2.01 (1.41–2.87)	0.0001
Pavlodar	2.95 (1.92–4.55)	0.0001	2.56 (1.64–4.01)	0.0001
North Kazakhstan	1.59 (1.04–2.43)	0.031	1.39 (0.89–2.18)	0.141
East Kazakhstan	3.63 (2.40–5.50)	0.0001	3.51 (2.29–5.36)	0.0001
Shymkent city	1 (Reference)	-	1 (Reference)	-
Smoking status				
Smokers	1.29 (1.09–1.51)	0.002	1.00 (0.83–1.19)	0.999
Non-smokers	1 (Reference)	—	1 (Reference)	—
Body Mass Index				
Underweight (<18.5 kg/m^2^)	1.24 (0.85–1.822)	0.249	1.17 (0.78–1.76)	0.441
Normal (18.5–24.99 kg/m^2^)	1.13 (0.99–1.28)	0.055	1.13 (0.98–1.30)	0.088
Overweight (≥25.0 kg/m^2^)	1 (Reference)	-	1 (Reference)	-

CI: confidence interval. OR: odds ratios.

## Data Availability

Data are available on reasonable request.

## Supplementary Materials

The following supporting information can be downloaded at: https://www.mdpi.com/article/10.3390/medicina61111913/s1, Table S1. STROBE checklist (cross-sectional).

## Abbreviations

The following abbreviations are used in this manuscript:

WHO	World Health Organization
GPAQ	Global Physical Activity Questionnaire
GPAQ-2	Global Physical Activity Questionnaire, version 2
STEPS	STEPwise Approach to Noncommunicable Disease Risk Factor Surveillance
GBD	Global Burden of Disease
DALY	Disability-Adjusted Life Years
MET	Metabolic Equivalent of Task
BMI	Body Mass Index
PHC	Primary Healthcare
PSU	Primary Sampling Unit
OR	Odds Ratio
CI	Confidence Interval
SPSS	Statistical Package for the Social Sciences
GCP	Good Clinical Practice
