# Supplementary material for: Physical Activity Levels in Kazakhstan: A Cross-Sectional Nationwide Study on Demographic, Socioeconomic, and Regional Factors
Source: Medicina (Kaunas). 2025 Oct 25;61(11):1913. doi: 10.3390/medicina61111913 (PMC12654467; doi:10.3390/medicina61111913)
Supplement: Supplementary file 1 [file medicina-61-01913-s001.zip › medicina-3903573-supplementary.pdf]

Supplementary

Table S1. STROBE checklist (cross-sectional)

Item	Recommendation (condensed)	Where reported
1. Title/Abstract	Indicate study design in title; provide informative, structured abstract.	Title; Abstract.
2. Background/Rationale	Explain scientific background and rationale.	Introduction, first paragraphs.
3. Objectives	State specific objectives (incl. prespecified hypotheses).	Introduction, final paragraph (primary/secondary objectives).
4. Study Design	Present key elements of design early.	2.1 Study Design.
5. Setting	Describe setting, locations, relevant dates (periods of recruitment/data collection).	2.1 Study Design (Kazakhstan; Oct 1, 2021–May 30, 2022).
6. Participants	Eligibility criteria; sources/methods of selection; sampling.	2.2 Sampling Design (multistage cluster; age 18–69; PSU/PHC/household selection).
7. Variables	Clearly define outcomes, exposures, predictors, confounders, effect modifiers.	2.4 Data Variables; 2.5 Physical Activity Measurement (PA categories, sedentary time; sociodemographics).
8. Data sources/Measurement	For each variable, give data sources and assessment methods.	2.3 Data Collection; 2.5 Physical Activity Measurement (GPAQ v2.0; WHO 2020; sedentary-time item).
9. Bias	Describe efforts to address potential sources of bias.	2.6 Bias; 6. Strengths and limitations (recall/social-desirability; no accelerometers; selection via PHC).
10. Study size	Explain how study size was arrived at.	2.2 Sampling Design (STEPS calculator; assumptions; n=6,585 target; n=6,720 recruited).
11. Quantitative variables	Explain handling of quantitative variables (groupings; rationale).	2.5 Physical Activity Measurement (MET-min/week; thresholds; category definitions); 2.7 Statistical Analysis.
12. Statistical methods	Describe all statistical methods, including those used to control for confounding; describe any methods used to examine subgroups and interactions; explain how missing data were addressed; if applicable, describe analytical methods taking account of the sampling strategy; describe any sensitivity analyses.	2.7 Statistical Analysis (adjusted logistic regression incl. all covariates).

13a. Participants	Report numbers at each stage.	2.2 + Figure 1 (n=6,720 recruited; n=6,587 analyzed; reasons for exclusion).
13b. Non-participation	Give reasons for non-participation at each stage.	Figure 1 (incomplete GPAQ domains; internal inconsistencies; implausible values).
13c. Flow diagram	Consider a flow diagram.	Figure 1. STROBE-compliant study flow diagram.
14. Descriptive data	Give characteristics of study participants (e.g., demographic, clinical, social) and information on exposures and potential confounders. Indicate number of participants with missing data for each variable of interest.	Results 3.1–3.6; Table 1–2.
15. Outcome data	Report numbers of outcome events or summary measures.	Results (prevalence not meeting WHO; PA categories; maps).
16a. Main results	Unadjusted and adjusted estimates with precision; confounders stated.	Table 5 (ORs, 95% CIs; variables adjusted).
16b. Category boundaries	Report category boundaries when continuous variables categorized.	2.5 (600 / 1,500 / 3,000 MET-min thresholds) and Tables.
16c. Translating estimates	Consider translating relative risk into absolute risk for a meaningful period.	Not applicable for cross-sectional ORs
17. Other analyses	Report subgroup analyses and interactions.	Results 3.1–3.6 (sex, age, region, ethnicity, BMI, smoking). Interactions not reported.
18. Key results	Summarize key results with reference to objectives.	Discussion.
19. Limitations	Discuss limitations (sources of bias/precision; direction and magnitude).	6. Strengths and limitations.
20. Interpretation	Cautious overall interpretation considering objectives and evidence.	Discussion (international frameworks; post-COVID; digital supports).
21. Generalisability	Discuss external validity of results.	Strengths and limitations (PHC-based recruitment and generalizability).
22. Funding (+ Ethics)	Sources of funding and role; Ethics approval and consent.	Funding; Institutional Review Board Statement (ethics; consent).
